# Enhancing Thin Film Properties of Chitosan–Collagen Biocomposites Through Potassium Silicate and Tannic Acid Integration

**DOI:** 10.3390/polym17050608

**Published:** 2025-02-25

**Authors:** Beata Kaczmarek-Szczepańska, Ugo D’Amora, Lidia Zasada, Marta Michalska-Sionkowska, Oliwia Miłek, Krzysztof Łukowicz, Anna Maria Osyczka

**Affiliations:** 1 Department of Biomaterials and Cosmetics Chemistry, Faculty of Chemistry, Nicolaus Copernicus University in Torun, Gagarin 7, 87-100 Toruń, Poland; 503555@doktorant.umk.pl; 2 Institute of Polymers, Composites and Biomaterials, National Research Council, v.le J.F. Kennedy 54, Mostra d’Oltremare Pad. 20, 80125 Naples, Italy; ugo.damora@cnr.it; 3 Department of Environmental Microbiology and Biotechnology, Faculty of Biological and Veterinary Sciences, Nicolaus Copernicus University in Toruń, Lwowska 1, 87-100 Toruń, Poland; mms@umk.pl; 4 Department of Cell Biology and Imaging, Institute of Zoology and Biomedical Research, Faculty of Biology, Jagiellonian University in Kraków, Gronostajowa 9, 30-387 Kraków, Poland; oliwiajmilek@gmail.com (O.M.); lukowicz.krzysiek@gmail.com (K.Ł.); anna.osyczka@uj.edu.pl (A.M.O.); 5 Competence Center for Periodontal Research, University Clinic of Dentistry, Medical University of Vienna, Sensengasse 2a, 1090 Vienna, Austria; 6 Laboratory of Immunoendocrinology, Department of Experimental Neuroendocrinology, Maj Institute of Pharmacology, Polish Academy of Sciences, 12 Smętna St., 31-343 Kraków, Poland

**Keywords:** biopolymers, tannic acid, potassium silicate, inorganic particles, surface properties

## Abstract

Chitosan and collagen are natural polymers widely used in biomaterials science; however, their inherent low stability and solubility present several challenges to obtain formulations suitable for potential clinical applications. In this study, tannic acid (TA) was employed as a cross-linker to improve the properties of thin films made from chitosan and collagen. In addition, potassium silicate (PS) was added as an inorganic filler, to produce innovative biocomposite films. The impact of TA and PS on physicochemical (i.e., material homogeneity, surface free energy, degradation, and stability roughness of surface), antioxidant, hemocompatibility, as well as cellular responses was evaluated. The results demonstrated that the incorporation of TA significantly enhanced the physicochemical properties of the chitosan/collagen-based films. The addition of 5% PS resulted in an increase in surface free energy and a decrease in roughness parameters. Furthermore, both surface free energy and cellular responses improved with the increased TA concentration in the biocomposite firms. Meanwhile, the hemolysis rate remained below 5%, indicating the potential suitability of these materials for medical applications, such as coatings or scaffolds for bone or skin wound healing.

## 1. Introduction

In recent years, materials science and engineering have significantly boosted the use of polymer blends and the design of micro/nanocomposites in the biomedical field [1,2]. Indeed, within the last three decades, there has been a noticeable rise in interest in novel materials based on polymer blends and composites [3,4,5,6]. In contrast to materials made of a single polymer, blends based on two or more biopolymers, either synthetic or natural, can allow the creation of a new class of materials with enhanced mechanical properties and biocompatibility. Similarly, benefiting from specific features of every single component, matrix, and structural filler (i.e., micro/nanoparticles, fibers), scientists have designed biomaterials with superior physicochemical, mechanical, and biological properties. The synergic combination of both strategies in the design of micro/nanocomposite polymer blends, i.e., biocomposite blends, has led to the development of a wide range of products, suitable for a variety of biomedical applications, such as tissue engineering, drug delivery, sensors, and implant design [2]. In tissue engineering, biocomposites have been used to create thin films, coatings, membranes, sponges, and scaffolds that can effectively recapitulate the structural properties of native tissues [7,8]. These biomaterials can provide a suitable milieu for cell growth and differentiation, promoting the development of functional living tissue substitutes. Furthermore, biocomposite-based drug delivery systems have been created to control the release of therapeutic drugs, improving efficacy and lowering toxicity [9]. Biocomposites have also been employed in the design of sensitive and selective biosensors for the detection of various biomolecules, such as proteins, enzymes, and nucleic acids [10], useful for disease diagnosis or drug monitoring. They have also demonstrated great promise in implant design and prosthetics, such as dental implants, cardiovascular stents, and joint replacements. These materials can display tailored mechanical and chemical properties, enhanced biocompatibility, a decreased risk of infection, and an improved ability to better integrate the device with the surrounding tissue [11,12].

Among the natural polymers, collagen has consistently attracted the greatest research attention. It serves as the primary structural component of all connective tissues, and it is the most common fibrous protein in the extracellular matrix (ECM). It has outstanding biocompatibility and is responsible oft issue stability [13]. To promote native tissue repair, it successfully supports the biological activity of cells [14]. With its triple helical peptide chain structure, collagen also controls several cell-material interactions that regulate intracellular signaling and eventually cell functions such as morphogenesis, ECM deposition, and tissue remodeling [5,15]. Its crucial function in the process of biomineralization has also been widely demonstrated [16]. In addition, chitosan and its derivatives have emerged as attractive polysaccharide candidates for biocomposites because of their remarkable biostimulative properties and good biocompatibility, cytocompatibility, and biodegradability [17]. Chitosan displays antibacterial and immunomodulatory properties. From a chemical point of view, it is similar to the glycosaminoglycans of the ECM [8]. Because of the above characteristics, chitosan is desirable for several tissue engineering applications [17]. It can provide structural support for cell adhesion, migration, and growth, as well as serve as a vehicle for controlled drug release to assist tissue survival and regeneration. Furthermore, several biomedical applications have been explored because of its exceptional film-forming features [13]. It is biocompatible and it forms complexes with other macromolecules, a variety of drugs, and proteins, including collagen, to generate biofunctional polymer blends [17,18]. Collagen and chitosan blends embedding micro/nanofillers have been extensively researched over the last years [19,20,21,22]. According to multiple research findings, both polymers are miscible in both solution and solid states. In addition, collagen fibers are not denatured when chitosan is added [23]. However, the collagen/chitosan weight ratio affects the blends’ thermal characteristics. Consequently, the studies on the miscibility, thermal, and rheological behavior of collagen/chitosan blends are presented [24]. Such blends also represent a good matrix for micro/nanofiller incorporation. For example, Becerra et al. developed collagen/chitosan composite membranes, incorporating hydroxyapatite (HAp) in different proportions (10 and 50% related to chitosan concentration) [13]. Collagen and HAp were added to chitosan to increase the thermal stability of the composites. It was demonstrated that HAp was well-dispersed in the organic matrix. The membranes with the highest HAp and collagen content provided good cell adhesion without cytotoxicity, indicating that these materials are promising for use in bone and skin tissue engineering [13]. The osteoregenerative potential of collagen/chitosan/HAp scaffolds was recently confirmed in vivo by Chacon et al. in defects created in healthy rats’ tibial bones, as well as in brittle bone following ovariectomy due to a lack of gonadal hormone [25]. All the rats showed evidence of bone development, but the non-ovariectomized groups, especially the ones treated with the scaffolds, showed a larger volume of bone formation, compared to the ovariectomized subgroups. Thus, these results demonstrated that the scaffolds were able to promote bone regeneration in vivo; however, aberrant conditions causing bone fragility due to gonadal hormone deprivation could lead to a delay in the repairing process. The authors also proved that altering the HAp synthesis conditions might be used to precisely control the rate and duration of bone growth [25]. By using a combination of chitosan/collagen/cerium HAp, Quilumbango et al. designed a drug delivery platform for in vitro gentamicin prolonged release. Compared to traditional immediate-release formulations, the results provided several benefits that might improve the antibacterial and therapeutic efficacy against Escherichia coli. In fact, after two hours, 80% of the gentamicin was released by the nanocomposites [26]. Collagen/chitosan blends have also been employed with silver nanoparticles (AgNPs) or zinc oxide nanoparticles (ZnO) [27,28,29]. For example, Sionkowska et al. obtained new composites based on collagen/chitosan films and AgNPs with antimicrobial properties [28]. AgNPs-containing collagen/chitosan displayed increased material’s tensile strength; films became less elastic and stiffer. Additionally, AgNPs increased the samples’ surface free energy. Finally, AgNP-containing collagen/chitosan films were found to be bacteriostatic against *Staphylococcus aureus* [28]. Recently, Zayed et al. developed ZnO loaded with propolis (PP/ZnO–NPs) that were embedded in marine collagen/chitosan gels [30]. ZnO-NPs’ surface negativity increased because of the addition of PP. The nanocomposite gel containing 10% PP/ZnO-NPs had the strongest inhibitory zone against *Escherichia coli* when tested for antibacterial activity. Surprisingly, in vivo results demonstrated remarkable tissue regeneration and collagen deposition by the nanocomposite gels [30].

Silicate nanoparticles have been widely employed to enhance the mechanical characteristics of both natural and synthetic polymers. Structure, modulus, strength, and toughness may be all significantly improved in the resulting polymer nanocomposites, all of which are not possible to accomplish by employing the polymer alone [31]. Furthermore, silica (SiO_2_) can be employed as a bone-replacing material. Indeed, it has been reported that silica is biocompatible and osteoconductive [32]. Different research groups have demonstrated that silicon is crucial for the synthesis of bone tissue and for the initial phases of mineralization in silica/collagen composites [32]. Indeed, silica-based biocomposites are bioactive due to the presence of a high density of surface silanol groups (Si-OH), which favor the formation of biologically active bone-like apatite [32]. Typically, silica precursors such as calcium silicate, sodium silicate, silicon catecholate, and orthosilicic acid are taken into consideration when creating silica-based biocomposites for bone tissue engineering [32].

Among them, bioceramics based on calcium silicate have been thoroughly studied and have demonstrated exceptional qualities [33]. Meanwhile, little is known about the use of potassium silicate (PS, SiO_2_:K_2_O) in the biomedical field.

PS may serve as a source of soluble silicon and potassium, containing approximately 34.5% SiO_2_ and 25% K_2_O [34]. It has been commonly used in the construction industry as a binder in the production of mineral paints and coatings, for industrial cleaning agents, or for the synthesis of technically advanced geopolymers [35,36]; in agriculture, as a supplement to improve plant health and resistance to diseases, pests, and weather conditions [37,38]. Notably, PS has been shown safe for the use in cosmetics and oral health [39]. The applications of PS are also explored in other fields, such as biomedical ones, where PS may allow for the development of more reliable products for bone health. The importance of potassium in the human body is widely recognized. The body’s ability to maintain an acid–base balance depends critically on potassium, which acts as an alkaline buffer, by neutralizing excess acids created during metabolic activities. The equilibrium between acid and base is closely linked to bone health [40]. Recent studies have clearly demonstrated that elevated blood acidity may lead to bone resorption. To counteract the acidity, the body releases calcium from bones, which may result in decreased bone density. Lastly, the synthesis of collagen by osteoblasts has been connected to potassium. Appropriate potassium levels are required for the deposition of collagen, which enhances the quality and integrity of bones [40].

To the best of our knowledge, this work represents the first attempt to design innovative biocomposites by using PS as an inorganic additive, to improve their overall physicochemical, mechanical, and biological properties. As a polymer matrix, we used collagen and chitosan, with the addition of PS, as the inorganic component. However, since natural polymers display low stability in an aqueous environment, tannic acid (TA) was added to the blend to improve its stability [41]. The PS and TA effects on the physicochemical properties of biocomposite films, their degradation and stability, as well as the response of bone marrow mesenchymal stem cells (BMSCs) have been investigated.

## 2. Materials and Methods

### 2.1. Chemicals

For film preparation: collagen (Coll) was isolated in our laboratory from the fish skin of Salmo salar [42]. Chitosan (CTS, low molecular weight, deacetylation degree (DD) = 77%), tannic acid (TA, M_w_ = 1701.2 g/mol), diiodomethane (99%), acetic acid (CH_3_COOH, ≥99.8%), phosphate buffer (PBS) solution, and absolute ethanol (>99.8%, EtOH) were supplied by Sigma Aldrich (Poznań, Poland). Potassium silicate (PS, anhydrous, −48 Mesh, SiO_2_:K_2_O 2.5:1 wt%) and 2,2-Diphenyl-1-picrylhydrazyl (DPPH, free radical, 95%) were obtained from Alfa Aesar (Karlsruhe, Germany). Glycerine (pure for analysis) was purchased from Avantor Performance Materials Poland S.A. (Gliwice, Poland).

For in vitro studies: phosphate-buffered saline (PBS) tablets were from BioShop Canada Inc. (Burlington, ON, Canada). Alpha- Minimum Essential Medium Eagle (MEM) was from Gibco by Thermo Fisher Scientific (Warsaw, Poland), Fetal Bovine Serum Qualified (FBS) from Biological Industries (Cromwell, CT, USA), and ZellShield from Minerva Biolabs (Berlin, Germany). MTS assay (CellTiter 96^®^ AQueous One Solution Cell Proliferation Assay) was from Promega (Madison, WI, USA).

### 2.2. Sample Preparation

Collagen and chitosan were dissolved in CH_3_COOH (0.1 M) at 1% concentration of both. TA was also dissolved in 0.1 M CH_3_COOH at a 2% concentration. Coll and CTS were mixed in a 50/50 *w*/*w*% ratio. Then, 5 and 20 *w*/*w*% of TA solution and 5 and 10 *w*/*w*% of PS powder were added. Polymer solutions with inorganic additives were stirred on the magnetic stirrer to obtain a homogeneous mixture. Then, the mixtures were placed on plastic covers for 48 h. Materials in thin film form were obtained by solvent evaporation. The obtained films were soft and ductile.

To assess the homogeneity of the obtained films, cross-section observations were conducted using a scanning electron microscope (SEM; LEO Electron Microscopy Ltd., Cambridge, UK). Prior to observation, the films were sputter-coated with gold.

### 2.3. Attenuated Total Reflect Fourier Transform Infrared (ATR-FTIR)

A Nicolet iS5 spectrophotometer (Thermo Fisher Scientific, Waltham, MA, USA) with an ID7 ATR attachment that featured a ZnSe crystal was used to obtain the films’ infrared spectra at room temperature in ambient air. Operating parameters included a wavenumber range of 4000 to 400 cm^−1^, 32 scans, and a resolution of 4 cm^−1^.

### 2.4. Contact Angle Measurement

The contact angle was determined by observing the liquid drop from the side [43]. The Owens–Wendt method [44] was used to determine the surface free energy (ɣ_s_), its polar (ɣ_SP_), and dispersive (ɣ_SD_) components from the contact angle measurement of two liquids: water and diiodomethane. A goniometer with a drop shape analysis system (DSA 10 Control Unit, Krüss, Hamburg, Germany) was used to perform the analysis at a constant temperature.

### 2.5. Swelling and Degradation Studies

For the swelling, studied films were immersed in PBS for 15, 30, 45, 60, 120, and 180 min. For the degradation study, the samples were immersed in a PBS solution for one week, and after three days, the PBS solution was changed to a fresh one. All containers were stored at 37 °C. The samples were weighed before and after the immersion and the degradation rate was calculated as the percentage of the weight change. Moreover, thickness before and after immersion was determined using a thickness gauge (Sylvac, Yverdon-les-Bains, Switzerland). The analysis was carried out in triplicate.

### 2.6. Atomic Force Microscopy (AFM)

The surface topography of the materials was analyzed using a NanoScope MultiMode SPM scanning probe microscope (Veeco Metrology, Inc., Santa Barbara, CA, USA) in tapping mode. Surface roughness parameters, including the root mean square (Rq) and arithmetic mean (Ra), were determined using NanoScope v6.11 software (Bruker Optic GmbH, Ettlingen, Germany).

### 2.7. DPPH Scavenging Assay

The antioxidant activity of the films was assessed using the DPPH reagent [45]. Film samples (1 cm^2^ area) were placed in a 12-well plate, and each well was filled with 2 mL of a 250 µM DPPH solution in methanol. The samples were kept in the dark for 1 h. Each test was conducted in triplicate. A DPPH solution left in the plate without any film sample served as the control. After the incubation period, the absorbance was measured at 517 nm using a spectrophotometer (UV-1800, Shimadzu Schweiz GmbH, Muttenz, Switzerland). The antioxidant activity (percentage of radical scavenging activity, RSA%) was then calculated using Equation (1):

(1) $$RSA\%=\frac{{Abs}_{DPPH}-{Abs}_{PB}}{{Abs}_{DPPH}}\times 100\%$$

where:

Abs_DPPH_ is the absorbance of the pure DPPH solution; Abs_PB_ is the absorbance of the DPPH solution in which the materials were immersed.

### 2.8. Blood Compatibility

Blood compatibility was assessed by using an adapted contact method, described by Zhou et al. [46]. A total of 10 mL of physiological saline solution containing different samples (1 cm^2^ area) was mixed with 0.2 mL of anticoagulated sheep blood. A total of 0.2 mL of fresh blood was added to water for the positive control and physiological saline for the negative one. The samples were incubated at 37 °C. After 1 h, the suspension was centrifuged for 10 min at 1000 rpm, and the supernatants were collected. The optical density ([OD]_specimen_), as well as [OD]_negative_ and [OD]_positive_ of all the supernatants, were measured at 540 nm by Multiscan FC spectrophotometer (Thermo Fisher Scientific, Waltham, MA, USA). The experiment was carried out three times in triplicate. The hemolysis rate was calculated using Equation (2):

(2) $$rate\;of\;hemolysis\;[\%]=\frac{\left[OD\right]specimen-\left[OD\right]negative}{\left[OD\right]positive-\left[OD\right]negative}\times 100\%$$

### 2.9. Material Sterilization for Cell Culture Studies

For in vitro cell studies, the materials were prepared in the form of thin films at the bottom of tissue culture plastic (TCP) plates. The films were disinfected with 75% EtOH for 10 min followed by rinsing with PBS to remove alcohol residues.

### 2.10. Assessing Cell Viability on Experimental Materials

Bone marrow mesenchymal stem cell (BMSC) cultures obtained from a 56-year-old male patient (Institutional Review Board Protocol Nr. 1072.6120.254.2017) were used. After the isolation, the cells were expanded in tissue culture flasks in a growth medium consisting of Alpha-MEM, 10% FBS and 1% ZellShield. The cells were detached from the culture flasks and seeded directly onto disinfected materials at a density of 1 × 10^4^/cm^2^ in 1 mL of growth medium in 24-well plates. The growth medium was exchanged every 2–3 days. MTS assay was performed to assess the metabolic activity of living cells. Briefly, after 6 days of cell culture, cells were rinsed with PBS and then supplemented with an MTS reagent diluted 10-fold in phenol-free Alpha-MEM. The diluted MTS was added to cell cultures in the amount of 200 μL per well/24-well plates. The reactions were developed in a CO_2_ incubator until the visible change in color in the culture wells vs. diluted MTS reagent in an empty well (blank). Afterwards, the MTS solution was transferred from the culture wells to separate wells in 96-well plates and its absorbance was measured at 492 nm using a plate reader (SpectraMax iD3 Molecular Devices, Molecular Devices, LLC, San Jose, CA, USA). According to the technical bulletin of CellTiter 96^®^ AQueous One Solution Cell Proliferation Assay, the intensity of the developed color is directly proportional to the amount of metabolically active cells.

### 2.11. Statistical Analysis

MTS tests for each type of material were performed in either triplicates or quadruplets. Values of MTS absorbance were averaged (mean value) and recalculated to a percentage change in the metabolic activity of the cells on the tested surfaces consisting of CTS, Coll, and either 5% or 10% PS with the addition of TA vs. cells viability on the materials without TA (assumed as 100%). The statistically significant differences were assessed with one-way ANOVA and post hoc Tukey test, and *p* < 0.05 was considered significant.

## 3. Results

### 3.1. Scanning Electron Microscope (SEM)

**Figure 1:**
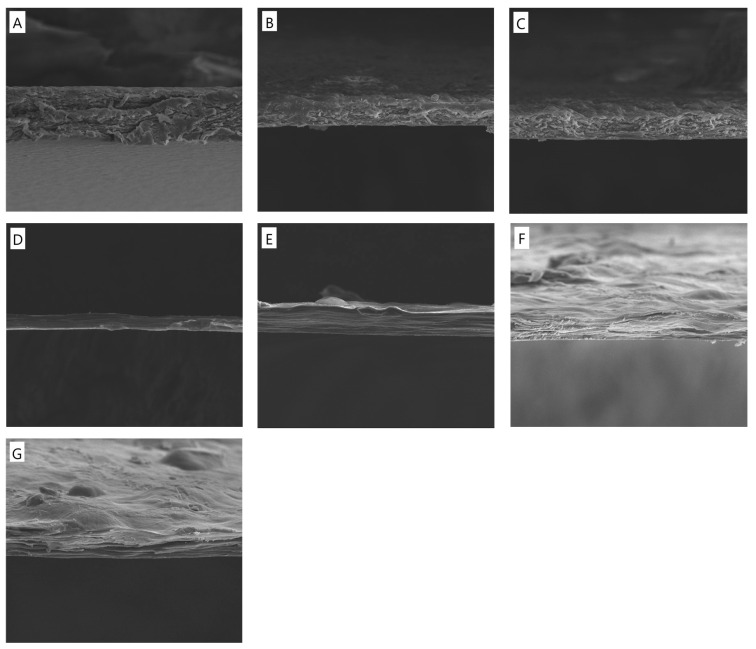
SEM images of cross-section morphology of the films based on (**A**) CTS/Coll, (**B**) CTS/Coll + 5%PS, (**C**) CTS/Coll + 10%PS, (**D**) CTS/Coll/5TA + 5%PS, (**E**) CTS/Coll/5TA + 10%PS, (**F**) CTS/Coll/20TA + 5%PS, (**G**) CTS/Coll/20TA + 10%PS (the presented images are representative for 3 specimens, mag. 2500×).

Based on the SEM images (Figure 1), the structural morphology of the different film formulations was analyzed to assess the impact of potassium silicate (PS) and tannic acid (TA) on the cross-section of the obtained materials. The control sample (CTS/Coll) exhibits a relatively uniform and porous structure with visible fibrous arrangements, suggesting good compatibility between chitosan (CTS) and collagen (Coll). The addition of potassium silicate (PS) at 5% and 10% concentrations leads to increased compaction in the polymer matrix. The structure appears denser, with reduced porosity compared to the control sample, indicating that PS enhances the material’s cohesion and mechanical stability. The introduction of tannic acid (TA) combined with potassium silicate results in noticeable structural changes. The network appears more compact, with fewer voids, suggesting enhanced crosslinking interactions between the polymer chains.

### 3.2. Attenuated Total Reflect Fourier Transform Infrared (ATR-FTIR)

**Figure 2:**
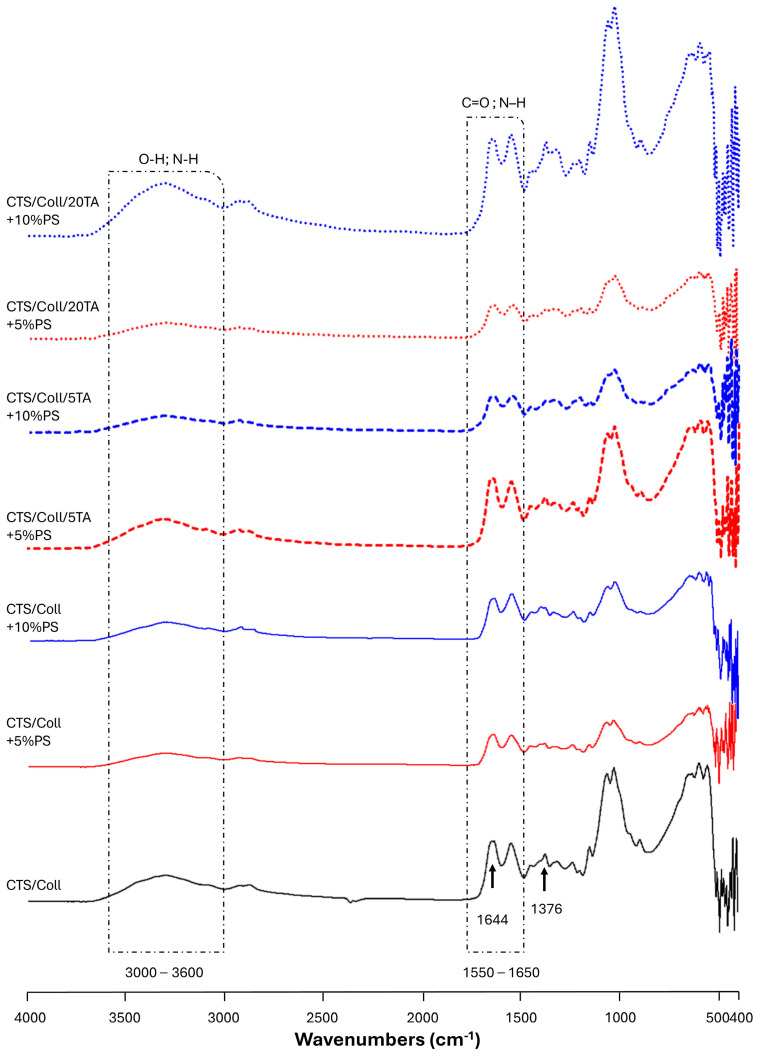
ATR-FTIR spectra of Chitosan/Collagen (CTS/Coll, black), CTS/Coll + 5% Potassium Silicate (PS) (red), and CTS/Coll + 10% PS (blue) films, uncross-linked and cross-linked by adding 5 or 20% tannic acid (TA), between 4000 and 400 cm^−1^. Uncross-linked films are indicated by a solid line; dashed lines indicate films cross-linked with 5% TA, while dotted lines indicate films cross-linked with 20% TA.

The principal functional groups of both polymers, chitosan, and collagen, may be identified using the vibrational bands shown by the ATR-FTIR spectra presented in Figure 2 and Figure S1. The broad band in the 3600–3000 cm^−1^ region is attributed to the axial deformations of the O–H and N–H groups, while the absorption bands at ~1544 cm^−1^ (–NH_2_), ~1644 cm^−1^ (amide I band), and ~1315 cm^−1^ (N-acetyl group) are typical of the polysaccharide structure. Additionally, the spectra display the distinctive absorption bands between 1550 and 1650 cm^−1^ (C=O and N–H stretching), which are attributed to collagen protein amides I (C=O stretching) and II (N–H bending and C–N stretching). However, for collagen detection, the strong signal always arises between 1700 and 1600 cm^−1^ [47]. In the present work, it is observed at ~1644 cm^−1^. In Figure 2, spectral bands associated with PS can also be detected near 1028 (Figure 2 and Figure S1). These bands are assigned to the asymmetric stretching of the Si–O–Si bond and OH vibration band of water and –SiOH groups, even though it is particularly challenging to analyze the bands due to the partial overlapping [48]. The characteristic bands of TA at 1703 cm^−1^ (C=O stretching) and 1173 cm^−1^ (C–O) are correlated to the aromatic esters (Figure S1). After the TA addition, slight changes were observed in the absorption bands. Indeed, the amide I of CTS/Coll + 10%PS passed from 1645 cm^−1^ to 1636 cm^−1^ (CTS/Coll/20TA + 10%PS). Meanwhile, no significant changes were observed for amide II in the presence of TA. The shifts in the major bands in the infrared spectra, particularly the amide I band of the CTS/Coll/TA blends, make it evident how the macromolecules interact, mostly through hydrogen bonds [41]. Indeed, TA can form hydrogen bonds with the chemical moieties found in collagen and chitosan.

### 3.3. Contact Angle Measurement

One of the most important parameters to take into consideration in implant tech-nology and tissue engineering is ensuring directed cell adhesion, which is strictly related to surface free energy. For this reason, surface free energy is often assessed as a measure of the ‘unsatisfied bond energy’ arising from ‘dangling bonds’ that are visible at the surface of a material. Protein adsorption, cell attachment, differentiation, and, ultimately, tissue formation at the interface, are strongly impacted by this unsatisfied bond energy [49,50]. Moreover, hydrophilicity must be determined because cells generally adhere better to hydrophilic surfaces as compared to hydrophobic ones [51,52]. The total surface energy of the specimens varied significantly depending on the composition (Table 1). The baseline CTS/Coll specimen had a surface energy of 26.93 mJ/m^2^. The introduction of 5% PS resulted in a slight decrease in surface energy to 26.56 mJ/m^2^, and this value remained almost unchanged with the addition of 10% PS (26.60 mJ/m^2^). However, the addition of TA caused a notable increase in surface energy. Specifically, the specimen with 5% TA and 5% PS showed an increase to 28.69 mJ/m^2^, and the addition of more TA (20%) led to a substantial increase in surface energy to 41.66 mJ/m^2^, indicating that TA significantly influences the overall surface energy.

The polar component of the surface energy was extremely low in the baseline CTS/Coll specimen (0.30 mJ/m^2^). When 5% PS was added, this component increased to 1.32 mJ/m^2^, suggesting a slight enhancement in polar interactions. However, with 10% PS, the polar component returned to its original low value (0.30 mJ/m^2^), indicating that the effect of PS on the polar component might be non-linear or dependent on other factors, such as concentration or interaction with other components. The introduction of TA significantly increased the polar component, with values of 3.63 mJ/m^2^ and 4.26 mJ/m^2^ for 5% and 10% PS, respectively, and further increased slightly with higher TA content, peaking at 4.76 mJ/m^2^. This indicates that TA contributes substantially to the polar interactions at the surface.

The dispersive component is the dominant contributor to the surface energy in all specimens. The baseline CTS/Coll had a dispersive energy of 26.63 mJ/m^2^, which slightly decreased with the addition of PS (both 5% and 10%). Interestingly, the introduction of TA, despite increasing the polar component, tended to decrease the dispersive component. For instance, in the CTS/Coll/5TA + 5%PS specimen, the dispersive energy dropped to 24.05 mJ/m^2^. This trend continues, with the lowest dispersive component observed in the CTS/Coll/20TA + 5%PS specimen (21.16 mJ/m^2^). This suggests that while TA enhances polar interactions, it might disrupt or alter the dispersive interactions, possibly due to changes in surface morphology or the chemical environment of the material.

### 3.4. Swelling and Degradation Studies

The low stability of natural-based polymers represents a primary limitation to their applications [53]. To address this issue, it is essential to modify the material composition to enhance their stability under physiological conditions. In this study, the degradation behavior of samples immersed in PBS was analyzed to evaluate their stability and the effects of compositional changes.

Table 2 presents the percentage of weight loss for various CTS/Coll specimens with different concentrations of potassium silicate (PS) and tannic acid (TA), alongside thickness measurements before and after immersion. Weight loss serves as a critical indicator of material degradation or dissolution, influenced by the composition and crosslinking within the specimens. Thickness measurements provide additional insights into potential swelling effects, helping to distinguish between degradation and structural expansion due to liquid absorption.

The CTS/Coll specimen without additives showed a weight loss of 38.69 ± 1.52%, indicating significant degradation, likely due to the absence of crosslinking agents or stabilizers. When 5% PS was added to the CTS/Coll matrix, the weight loss decreased to 25.01 ± 2.97%, suggesting that PS enhances material stability by improving intermolecular interactions and resistance to degradation.

The introduction of TA also had a substantial effect on weight loss. For example, the CTS/Coll/5TA + 5%PS specimen exhibited a weight loss of 34.52 ± 2.02%, slightly lower than the CTS/Coll control but higher than CTS/Coll + 5%PS. At lower concentrations (5%), TA provided some degree of crosslinking, although its stabilizing effect was less pronounced compared to PS. At higher TA concentrations (20%), the reduction in weight loss became significant. The CTS/Coll/20TA + 5%PS specimen showed a weight loss of 5.26 ± 0.56%, while CTS/Coll/20TA + 10%PS exhibited a similar weight loss of 5.88 ± 0.27%. These findings suggest that TA acts as a robust crosslinking agent at higher concentrations, significantly enhancing stability and reducing susceptibility to degradation.

The synergistic effect of PS and TA was particularly evident at higher TA concentrations. While PS alone reduced weight loss to some extent, the addition of TA at 20% led to a pronounced reduction in degradation. This demonstrates that PS stabilizes the matrix by improving intermolecular interactions, while TA provides strong crosslinking, resulting in significantly enhanced stability.

**Figure 3:**
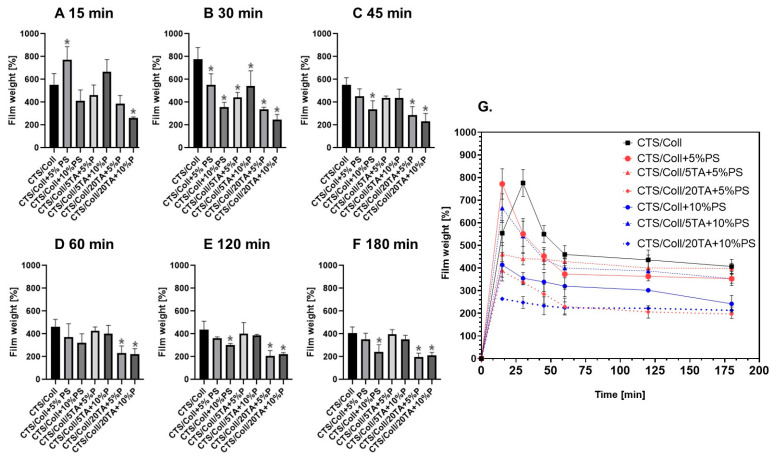
The films’ weight changes after immersion in PBS for (**A**) 15, (**B**) 30, (**C**) 45, (**D**) 60, (**E**) 120, and (**F**) 180 min. (**G**) Curves up to 180 min highlighting the swelling *plateau* effect (n = 3; * significantly different from CTS/Coll—*p* < 0.05).

The films’ weight after immersion in PBS for 15, 30, 45, 60, 120, and 180 min demonstrated significant changes, reflecting the dynamic behavior of the polymer matrix in aqueous environments (Figure 3). These changes highlight the combined effects of swelling and degradation during immersion.

At 15 min, CTS/Coll films showed an initial increase in weight, likely due to water absorption. Films containing 5% PS exhibited the highest swelling, as indicated by their significant weight gain compared to the control. In contrast, films with high concentrations of TA (20%) and PS demonstrated reduced swelling, suggesting that the crosslinking effects of TA and the stabilizing role of PS limited water uptake.

By 30 and 45 min, the swelling effect reached a *plateau* for most samples, particularly for films with higher TA concentrations. While the CTS/Coll control and low-PS specimens maintained higher weights, specimens with 20% TA showed substantially reduced weight gains. This highlights the ability of TA to mitigate excessive swelling by enhancing the structural integrity of the films.

At 60 and 120 min, weight changes stabilized further, with degradation effects becoming more apparent in samples with lower crosslinking. Films with high TA concentrations (20%) retained their weight more effectively, indicating their resistance to degradation. This contrasts with the CTS/Coll control, which showed noticeable weight loss during these time points.

At 180 min, films with 20% TA (CTS/Coll/20TA + 5%PS and CTS/Coll/20TA + 10%PS) were the most stable among the tested samples. The reduction in weight observed in the control and low-PS specimens reflects material degradation, while the stabilized weight of high-TA films underscores the protective effects of strong crosslinking.

Thickness measurements before and after immersion further validate these findings. The CTS/Coll control experienced a marked decrease in thickness, consistent with substantial degradation. Specimens with added PS exhibited reduced thickness loss, indicating improved stability. The addition of TA further minimized thickness reductions, particularly at higher concentrations (20%), where specimens exhibited minimal changes in thickness. This demonstrates that TA reinforces the structural integrity of the matrix, mitigating both degradation and swelling effects during immersion.

The combined analysis of weight loss and thickness changes reveals that the addition of PS and TA synergistically stabilizes the CTS/Coll matrix. PS improves stability by enhancing intermolecular interactions, while TA provides robust crosslinking, particularly at higher concentrations. These findings confirm that the combination of PS and TA effectively reduces degradation and mitigates swelling, making the materials more suitable for physiological conditions.

### 3.5. Atomic Force Microscopy (AFM)

**Figure 4:**
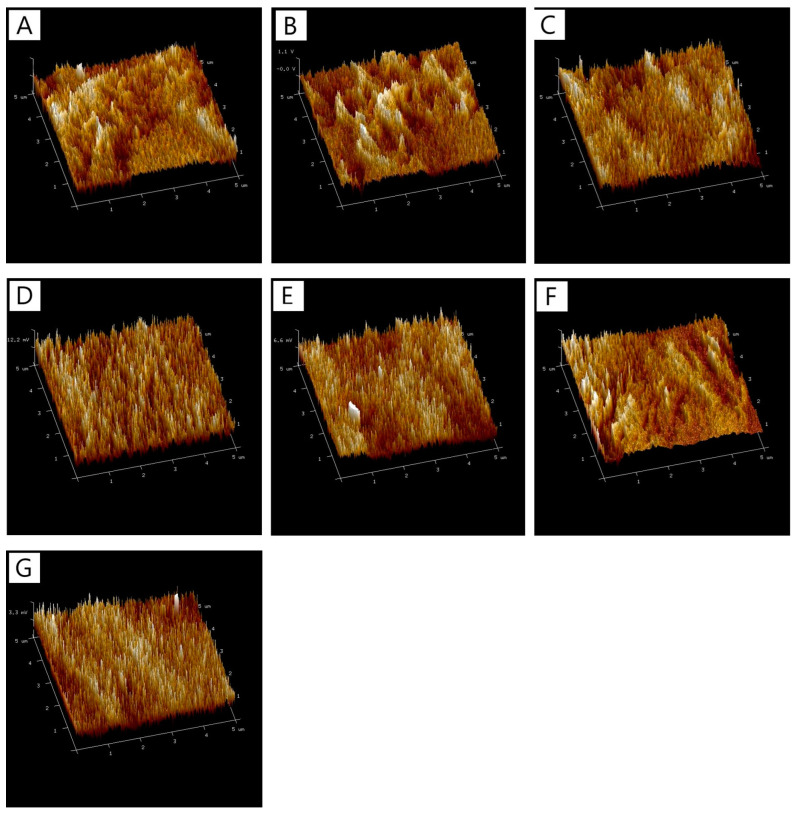
The 3D images of surfaces of films (**A**) CTS/Coll, (**B**) CTS/Coll + 5%PS, (**C**) CTS/Coll + 10%PS, (**D**) CTS/Coll/5TA + 5%PS, (**E**) CTS/Coll/5TA + 10%PS, (**F**) CTS/Coll/20TA + 5%PS, (**G**) CTS/Coll/20TA + 10%PS.

The analysis of surface roughness parameters, including the arithmetic mean roughness (Ra) and root mean square roughness (Rq), revealed no significant differences in these values across most of the samples studied (Figure 4). Table 3 summarizes the roughness parameters (Ra and Rq) for CTS/Coll films with varying compositions, showing a narrow range of variability.

The base CTS/Coll sample exhibited Ra and Rq values of 4.40 ± 0.88 nm and 5.57 ± 1.16 nm, respectively. The addition of 5% and 10% PS to the CTS/Coll matrix slightly increased the roughness values (e.g., CTS/Coll + 10%PS had Ra = 5.22 ± 1.93 nm and Rq = 6.92 ± 3.88 nm), suggesting that the inclusion of PS did not drastically alter the surface morphology. These minor changes may be attributed to the dispersion of PS within the matrix, which does not significantly impact the overall surface texture at the nanoscale level.

Interestingly, the incorporation of tannic acid (TA) alongside PS resulted in slightly larger variability in Ra and Rq values, though still within the same general range as the base material. For example, CTS/Coll/20TA + 5%PS had Ra and Rq values of 3.18 ± 1.39 nm and 3.68 ± 1.23 nm, respectively, which were slightly higher compared to the control. These results suggest that TA at higher concentrations may introduce localized roughness due to its crosslinking effect. However, for samples with 20% TA and 10% PS (CTS/Coll/20TA + 10%PS), the Ra and Rq values were the lowest among all tested samples (Ra = 2.40 ± 0.82 nm, Rq = 2.93 ± 1.18 nm), indicating that this combination creates a smoother surface.

### 3.6. DPPH Scavenging Assay

The antioxidant activity of the films was determined, and the radical scavenging activity (RSA%) is shown in Table 3. The RSA values indicate the antioxidant capacity of the materials, with higher percentages signifying a greater ability to neutralize free radicals. The CTS/Coll specimen without any additives exhibited an RSA of 22.01 ± 3.40%. This value provides a baseline for comparison, reflecting the inherent antioxidant activity of the CTS/Coll matrix. The addition of 5% PS slightly reduced the RSA to 21.90 ± 0.80%, and further increasing the PS concentration to 10% resulted in a slight additional decrease to 21.30 ± 0.70%. These changes are minimal and suggest that PS does not significantly impact the antioxidant capacity of the material.

The introduction of TA led to a substantial increase in RSA across all specimens, indicating a significant enhancement in antioxidant activity. For instance, the specimen with 5% TA and 5% PS demonstrated an RSA of 53.50 ± 0.47%, more than double the antioxidant capacity compared to the baseline CTS/Coll. Further increasing the TA concentration to 20% resulted in the highest RSA values recorded: 61.45 ± 0.23% for the specimen with 5% PS, and 60.91 ± 0.13% for the specimen with 10% PS. The marginal difference between these two indicates that while TA is the dominant factor in enhancing RSA, the concentration of PS has a minimal effect on the antioxidant capacity when TA is present in excessive amounts.

### 3.7. Blood Compatibility

Because of the shear stress, erythrocytes, the blood’s most rigid cells, are susceptible to hemolysis. The ASTM F756-00 standard classifies materials as non-hemolytic if their hemolytic index is between 0 and 2%, somewhat hemolytic if their index is between 2 and 5%, and hemolytic if their index is less than 5% [54]. The results of blood compatibility tests are listed in Table 4. The hemolysis of the materials studied was in the range of 0.03 to 0.21%. The 10% PS addition resulted in an increased rate of hemolysis. The presence of TA decreased the hemolysis rate, probably due to the increased material biocompatibility. It can be assumed that each type of specimen showed a rate of hemolysis below 5%.

### 3.8. Cell Response

**Figure 5:**
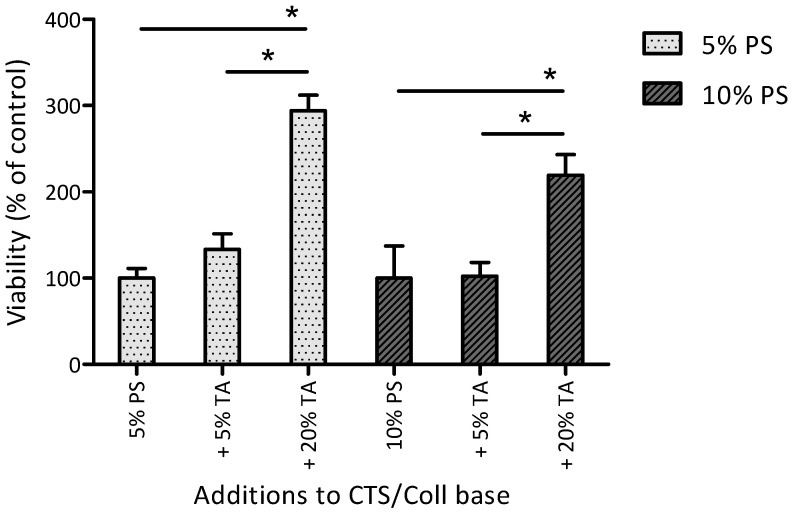
Metabolic activity of BMSC cultured for 6 days on different material films. The results are reported as mean STD (i.e., % change in BMSC metabolic activity on the material surface vs. material control without TA). CTS/Coll films combined in the ratio of 50:50 (*w*/*w*%) with the addition of either 5% or 10% PS and 5% or 20% TA. * Statistically significant differences between 5% PS and 10% PS groups.

Materials prepared of CTS and Coll combined at the ratio of 50:50 *w*/*w*% with the addition of either 5% or 10% PS showed similar effects on BMSC metabolic activity (Figure 5). The addition of 5% TA to the materials containing 5% PS (gray bars) slightly (but not statistically significantly) increased BMSC metabolic activity, whereas the addition of 5% TA to the materials containing 10% PS (black bars) did not affect BMSC metabolic activity. In contrast, the addition of 20% TA significantly increased BMSC metabolic activity: by 194 ± 18% in materials containing 5% PS and by 119 ± 24% in materials containing 10% PS.

## 4. Discussion

Chitosan/Collagen (CTS/Coll) and biocomposite films, by embedding potassium silicates (PS) as an inorganic matrix, were successfully produced by the solvent casting method. The addition of potassium silicate (PS) enhances film compactness by reducing porosity, while tannic acid (TA) strengthens the matrix through crosslinking interactions. The highest concentrations of both components (20% TA + 10% PS) result in the most compact and stable structure. The films were cross-linked by using different concentrations of tannic acid (5 and 20% TA). ATR-FTIR analysis was used to identify the functional groups in the films (Figure 2). Furthermore, by comparing the spectra (red or blue curves, in Figure 1) it is possible to assess the effect of TA functionalization. Indeed, small changes were observed in the amide I peak positions after the addition of TA, ascribable to possible interactions between the macromolecules and TA, mostly through hydrogen bonds [41].

The results clearly demonstrate that TA significantly influences the surface properties of CTS/Coll-based materials. The observed increase in total surface energy and the polar component with the addition of TA indicates that TA effectively enhances the hydrophilicity and polar interactions on the material’s surface. This enhancement is likely due to the high density of hydroxyl groups (OH) in TA, which can form hydrogen bonds and contribute to the increased polar surface energy, as supported by similar findings in the literature [55].

However, the concurrent decrease in the dispersive component suggests a trade-off between enhancing polar interactions and maintaining dispersive forces, which are typically associated with van der Waals interactions. This phenomenon has been observed in other studies, where the introduction of polar groups altered the balance of surface energy components, leading to a decrease in the dispersive energy [56].

The addition of PS, although less impactful than TA, also modifies the surface properties, but its effects appear to be concentration-dependent and non-linear. This behavior could be attributed to the role of PS in forming siloxane bonds, which might contribute to the structural rearrangement of the surface but do not uniformly enhance polar interactions. This result is consistent with previous research, in which the inclusion of silicate in polymer matrices influenced surface characteristics in a non-linear fashion, depending on the concentration and interaction with another component [57].

These findings have significant implications for the potential applications of these materials, especially in areas where surface interactions, such as adhesion, wettability, and biocompatibility, are critical. For instance, in biomedical applications such as wound healing, the ability to fine-tune the surface energy components by adjusting the TA and PS content could lead to materials with optimized cell adhesion properties and enhanced biocompatibility. Similarly, the results of this work could be easily exploited in bone or skin wound healing or other industrial applications where specific surface characteristics are required, such as coatings or adhesives. The tunability of these materials could offer tailored solutions for different substrates and environmental conditions.

TA, however, demonstrates a much more pronounced effect on the stability of the materials, particularly at higher concentrations as evidenced by the significant reduction in weight loss during degradation. This substantial reduction in degradation can be attributed to the formation of strong hydrogen bonds between TA and the biopolymer chains, which has been well-documented in the literature as an effective strategy for enhancing the durability and longevity of biomaterials [58].

The implications of these findings are particularly relevant for the design and development of biopolymer materials intended for biomedical applications. In scenarios where material stability is of paramount importance, such as in biomedical implants, or other long-term use devices, the ability to fine-tune the degradation rate by adjusting the concentrations of PS and TA is invaluable. For instance, in applications requiring rapid degradation, such as temporary scaffolds for tissue engineering, lower concentrations of TA might be used to ensure timely resorption. Conversely, in applications where long-term stability is desired, such as in permanent implants, higher concentrations of TA can be employed to significantly extend the material’s lifespan [59].

The lack of significant differences in Ra and Rq across most samples highlights that surface roughness is not substantially altered by the inclusion of PS and TA in the CTS/Coll matrix. This suggests that the observed changes in other properties, such as stability and degradation resistance, are more likely related to chemical or structural changes within the bulk material rather than surface morphology. However, it is worth noting that the smoother surfaces observed for CTS/Coll/20TA + 10%PS may contribute to enhanced material properties, such as better interaction with surrounding environments or reduced surface susceptibility to degradation. Similar results were obtained by Halim et al. [60], where tannic acid reduced the roughness of chitosan-based film.

Overall, while the roughness parameters Ra and Rq show minor variability, they remain consistent enough to conclude that surface morphology does not undergo drastic alterations with the tested modifications in material composition. These findings further support the notion that bulk material changes, such as crosslinking and chemical stabilization, play a more critical role in determining the performance of these polymer films.

The relatively low RSA of CTS/Coll films suggests that the base material has limited ability to scavenge free radicals, which is consistent with previous studies that have shown chitosan and collagen to possess some, but not significant, antioxidant properties [61]. The addition of 5% PS slightly reduced the RSA. This aligns with previous findings where silicate-based additives were observed to have a negligible effect on the antioxidant properties of composite materials [62]. The increase in RSA after the addition of TA to the material can be attributed to the strong antioxidant properties of TA, which is known for its ability to donate hydrogen atoms and neutralize free radicals effectively [63]. The results suggest that TA’s role as an antioxidant is both potent and concentration-dependent, significantly boosting the material’s ability to scavenge free radicals as its concentration increases.

The significant increase in RSA with the addition of TA highlights its potential as a functional additive in CTS/Coll-based materials, particularly for applications where antioxidant properties are crucial, where oxidative stress can impede the regeneration process. The ability to enhance the antioxidant capacity of these materials through the inclusion of TA could make them particularly suitable for biomedical applications that require protection against oxidative damage [59].

Additionally, the results suggest that the combination of TA and PS can be optimized to tailor the antioxidant properties of the material, depending on the specific requirements of the application. For instance, materials with higher TA content could be developed for applications that demand higher antioxidant activity, while those with lower TA might be suitable for environments where less radical scavenging is needed.

The presence of TA in the materials appeared to decrease the hemolysis rate, which might be attributed to the enhanced biocompatibility conferred by TA. Tannic acid is known for its antioxidant and anti-inflammatory properties, which could contribute to reducing the stress on erythrocytes and thus lower the hemolysis rate. This finding aligns with the results of a study by Harpe et al., which reported that tannic acid-modified materials exhibited improved blood compatibility due to their ability to reduce oxidative stress and stabilize cell membranes [64]. Further supporting evidence comes from research conducted by Argenziano et al., who demonstrated that tannic acid incorporation into polymeric films significantly reduced hemolytic activity, attributed to the compound’s ability to protect cell membranes from oxidative damage [65].

In the study by Chen et al., TA significantly enhanced cell proliferation and metabolic activity when used as a crosslinking agent in biopolymer matrices, consistent with our results obtained for materials containing 20% TA that showed substantially increased BMSC metabolic activity with either 5% or 10% PS [66]. In contrast to TA, PS did not significantly influence BMSC metabolic activity. Notably, in materials containing 20% TA, PS at 10% seemed to lower BMSC metabolic activity vs. 5% concentration. Thus, for cellular biocompatibility, optimization of the TA/PS ratio may be necessary.

## 5. Conclusions

Composite materials with polymeric matrix and inorganic additives provide many advantages. Inorganic particles improve the surface properties of polymer-based films. The increase in surface free energy and decrease in roughness parameters were relevant to the increase in cell viability on the materials after the addition of low potassium silicate amounts. Also, the increase in tannic acid content resulted in an increase in surface free energy and the cells’ viability. All the tested films showed a hemolysis rate below 5%. It can be assumed that potassium silicate and tannic acid additives provide overall improvement of chitosan/collagen-based materials, but optimization of the TA/PS ratio in such biocomposites is necessary to obtain good biocompatibility with BMSC cells.

## Figures and Tables

**Table 1:** Surface free energy (ɣs), its polar (ɣsP) and dispersive (ɣsD) components of thin films based on chitosan (CTS) and collagen (Coll) with tannic acid (TA) and potassium silicate (PS) (n = 5; * significantly different from CTS/Coll—*p* < 0.05).

Specimen	ɣs [mJ/m^2^]	ɣsP [mJ/m^2^]	ɣsD [mJ/m^2^]
CTS/Coll	26.93 ± 0.40	0.30 ± 0.04	26.63 ± 0.36
CTS/Coll + 5%PS	26.56 ± 0.31	1.32 ± 0.04 *	25.24 ± 0.27 *
CTS/Coll + 10%PS	26.60 ± 0.11	0.30 ± 0.01	26.30 ± 0.10
CTS/Coll/5TA + 5%PS	28.69 ± 0.26 *	3.63 ± 0.06 *	24.05 ± 0.20 *
CTS/Coll/5TA + 10%PS	27.63 ± 0.33 *	4.26 ± 0.07 *	25.37 ± 0.26 *
CTS/Coll/20TA + 5%PS	41.66 ± 0.27 *	4.50 ± 0.14*	21.16 ± 0.14 *
CTS/Coll/20TA + 10%PS	30.17 ± 0.31 *	4.76 ± 0.17 *	24.90 ± 0.13 *

**Table 2:** The weight loss after the degradation in PBS and thickness of films based on chitosan (CTS) and collagen (Coll) with tannic acid (TA) and potassium silicate (PS) before and after immersion in PBS films (n = 3; * significantly different from CTS/Coll—*p* < 0.05).

Specimen	Weight Loss [%]	Thickness Before [mm]	Thickness After [mm]
CTS/Coll	38.69 ± 1.52	0.0867 ± 0.0038	0.0563 ± 0.0014
CTS/Coll + 5%PS	25.01 ± 2.97 *	0.0730 ± 0.0043 *	0.0513 ± 0.0038
CTS/Coll + 10%PS	21.34 ± 2.11 *	0.0607 ± 0.0029 *	0.0473 ± 0.0014
CTS/Coll/5TA + 5%PS	34.52 ± 2.02	0.0827 ± 0.0052 *	0.0557 ± 0.0029
CTS/Coll/5TA + 10%PS	19.08 ± 1.83 *	0.0643 ± 0.0052 *	0.0527 ± 0.0038
CTS/Coll/20TA + 5%PS	5.26 ± 0.56 *	0.0750 ± 0.0090	0.0687 ± 0.0076 *
CTS/Coll/20TA + 10%PS	5.88 ± 0.27 *	0.0690 ± 0.0025 *	0.0640 ± 0.0025

**Table 3:** The roughness parameters (Ra and Rq) and RSA of films based on chitosan (CTS) and collagen (Coll) with tannic acid (TA) and potassium silicate (PS) (n = 5; * significantly different from CTS/Coll—*p* < 0.05).

Specimen	Ra [nm]	Rq [nm]	RSA [%]
CTS/Coll	4.40 ± 0.88	5.57 ± 1.16	22.01 ± 3.40
CTS/Coll + 5%PS	5.34 ± 0.58	6.65 ± 0.11	21.90 ± 0.80
CTS/Coll + 10%PS	5.22 ± 1.93	6.92 ± 3.88	21.30 ± 0.70
CTS/Coll/5TA + 5%PS	5.16 ± 1.60	6.37 ± 1.91	53.50 ± 0.47 *
CTS/Coll/5TA + 10%PS	3.41 ± 0.33	4.28 ± 0.12	54.10 ± 0.50 *
CTS/Coll/20TA + 5%PS	3.18 ± 1.09	3.68 ± 1.23	61.45 ± 0.23 *
CTS/Coll/20TA + 10%PS	2.40 ± 0.82	2.93 ± 1.18	60.91 ± 0.13 *

**Table 4:** The rate of hemolysis for the material samples prepared from chitosan (CTS) and collagen (Coll) with tannic acid (TA) and potassium silicate (PS) (n = 3; * significantly different from CTS/Coll—*p* < 0.05).

Specimen	Rate of Hemolysis [%]
CTS/Coll	0.04 ± 0.01
CTS/Coll + 5%PS	0.03 ± 0.01
CTS/Coll + 10%PS	0.21 ± 0.07 *
CTS/Coll/5TA + 5%PS	0.04 ± 0.01
CTS/Coll/5TA + 10%PS	0.03 ± 0.01
CTS/Coll/20TA + 5%PS	0.16 ± 0.01 *
CTS/Coll/20TA + 10%PS	0.17 ± 0.06 *

## Data Availability

The original contributions presented in this study are included in the article/Supplementary Materials. Further inquiries can be directed to the corresponding author.

## Supplementary Materials

The following supporting information can be downloaded at: https://www.mdpi.com/article/10.3390/polym17050608/s1, Figure S1: ATR-FTIR spectra of Chitosan (CTS, Solid black line), Collagen (Coll, dashed black line), potassium silicate (PS, solid red line) and tannic acid (TA, dashed red line) between 4000 and 400,650 cm^−1^.
