# Supplementary material for: Enhancing Thin Film Properties of Chitosan–Collagen Biocomposites Through Potassium Silicate and Tannic Acid Integration
Source: Polymers (Basel). 2025 Feb 25;17(5):608. doi: 10.3390/polym17050608 (PMC11902633; doi:10.3390/polym17050608)
Supplement: Supplementary file 1 [file polymers-17-00608-s001.zip › polymers-3473962-supplementary.pdf]

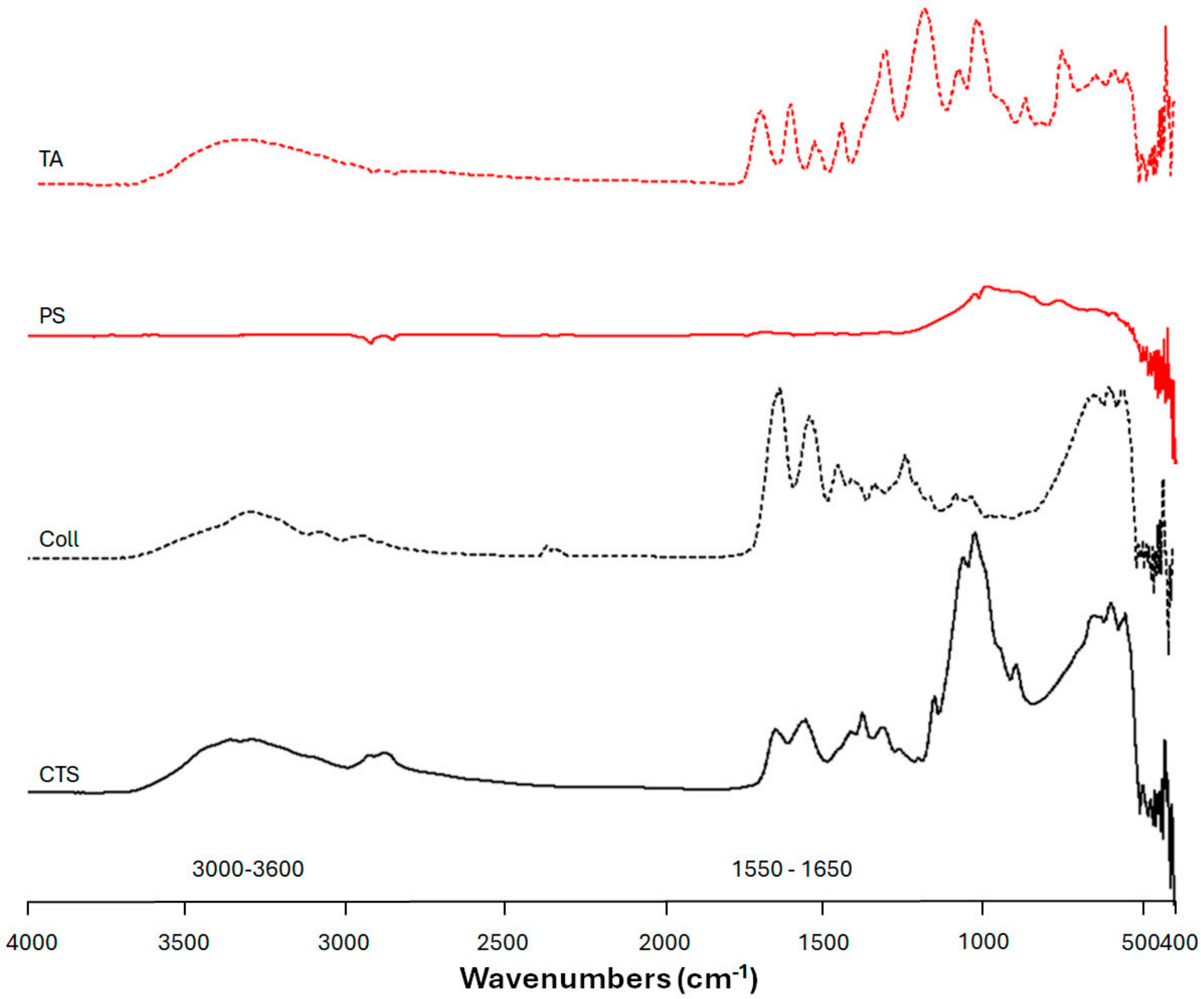


**Figure S1**. ATR-FTIR spectra of Chitosan (CTS, Solid black line), Collagen (Coll, dashed black line), potassium silicate (PS, solid red line) and tannic acid (TA, dashed red line) between 4000 and 400650 cm-1.
